# Geometrothermodynamics of 3D Regular Black Holes

**DOI:** 10.3390/e26060457

**Published:** 2024-05-28

**Authors:** Nurzada Beissen

**Affiliations:** Institute for Experimental and Theoretical Physics, Al-Farabi Kazakh National University, Almaty 050040, Kazakhstan; nurzada.beissen@kaznu.edu.kz

**Keywords:** geometrothermodynamics, black hole, equilibrium thermodynamics

## Abstract

We investigate a spherically symmetric exact solution of Einstein’s gravity with cosmological constant in (2 + 1) dimensions, non-minimally coupled to a scalar field. The solution describes the gravitational field of a black hole, which is free of curvature singularities in the entire spacetime. We use the formalism of geometrothermodynamics to investigate the geometric properties of the corresponding space of equilibrium states and find their interpretation from the point of view of thermodynamics. It turns out that, as a result of the presence of thermodynamic interaction, the space of equilibrium states is curved with two possible configurations, which depend on the value of a coupling constant. In the first case, the equilibrium space is completely regular, corresponding to a stable thermodynamic system. The second case is characterized by the presence of two curvature singularities, which are shown to correspond to locations where the system undergoes two different phase transitions, one due to the breakdown of the thermodynamic stability condition and the second one due to the presence of a divergence at the level of the response functions.

## 1. Introduction

General relativity is widely accepted as the best candidate to describe the gravitational field as a fundamental interaction. An important prediction of general relativity is the prediction of the existence of a particular set of exact solutions, black holes, in which a local curvature singularity is surrounded by an event horizon that does not permit sending information from the interior of the horizon to the exterior spacetime. Recently [1,2,3,4,5,6], certain gravitational effects have been observed directly, pointing to the existence of black hole configurations in the universe.

The presence of curvature singularities in the theory of general relativity has been considered for decades as an indication of the breakdown of the theory, which can be corrected by including a new theory probably related to quantum gravity—a theory still under construction [7]. This does not mean that general relativity should be discarded as a theory of the gravitational field. In fact, it can be applied to describe any effects in which gravity can be considered a classical (non quantum) interaction. The appearance of singularities indicates that general relativity should be complemented in such a way that it can also describe gravitational effects at the quantum level.

On the other hand, a less challenging proposal consists of replacing the singularities with regular regions filled with matter, implying modifications of classical general relativity. Such configurations are known as regular black holes and have been proposed as alternative sources for the gravitational field of compact objects. One of the first examples of a regular black hole [8] utilized non-linear electrodynamics as matter source. In particular, a magnetic monopole has been used as the matter distribution that covers the curvature singularity [9,10], leading to a gravitational configuration free of curvature singularities in the entire spacetime.

Regular black holes are also known to exist in (2 + 1) dimensions [11]. In particular, configurations with non-linear electrodynamics as an additional matter source have been extensively investigated [12,13,14,15,16,17]. In the present work, we will study a 3D black hole that contains as its additional matter sources a cosmological constant and a scalar field. Our study of this particular black hole will focus on the investigation of its thermodynamic properties from the point of view of the theory of geometrothermodynamics (GTD). This theory is invariant with respect to Legendre transformations, which are used in classical thermodynamics to relate different thermodynamic potentials. This means that classical thermodynamics and GTD are both independent of the choice of thermodynamic potential. For explicit computations, this is a very important property because it happens very often that a specific thermodynamic potential is known only in a particular representation, and a transformation into another potential cannot be carried out explicitly.

This paper is organized in the following form. In Section 2, we present the explicit form of the 3D action and the solution, which includes a cosmological constant and a scalar field. In Section 3, we review the classical thermodynamics methods used to describe a given thermodynamic system. In Section 4, we derive the Legendre invariant metrics of the phase space and the space of equilibrium states that are used in GTD to represent any thermodynamic system in terms of differential manifolds. Then, in Section 5, we study the geometric properties of the space of equilibrium states and find that there are two curvature singularities. In Section 6, we show that the curvature singularities correspond to thermodynamic critical points, where the black hole passes into a state of non-equilibrium accompanied by phase transitions.

## 2. Static Regular Black Holes

The Banados–Teitelboim–Zanelli (BTZ) spacetime represents the simplest black hole solution in (2 + 1)-dimensional gravity [18]. It is a solution of Einstein’s equations in which the cosmological constant Λ acts as the source of gravity, i.e., the Einstein–Hilbert action is given as $S_{EH}=\int d^{3}x\sqrt{-g}\left(R+2L\right)$, where $L=-\Lambda =-1/l^{2}$, with *l* being the curvature radius. The cosmological constant is negative so that *L* is positive definite (for a review, see [11]). One important property of this spacetime is that it is completely free of curvature singularities.

The BTZ solution was the starting point for the derivation of other regular spacetimes in 3D gravity. To this end, the main approach consists of considering additional terms in the Einstein–Hilbert action of the form

(1) $$S_{EH}=\int d^{3}\sqrt{-g}\left(R+2L+L_{m}\right),$$

where $L_{m}$ represents a matter Lagrangian. In this work, we will consider a scalar field φ, which is non-minimally coupled to gravity, as expressed in the Lagrangian

(2) $$L_{m}=\frac{1}{L}\left\{{\left(\partial \varphi \right)}^{4}-\sqrt{\left|\xi \right|}\left[3R^{\mu \nu }\partial_{\mu }\varphi \partial_{\nu }\varphi -R{\left(\partial \varphi \right)}^{2}\right]\right\}.$$

We see that, in this particular Lagrangian, the cosmological constant also plays the role of coupling constant together with ξ.

For the particular solution we will investigate in this work, the spacetime geometry in spherical-like coordinates (t,r,ϕ) can be expressed as

(3) $$ds^{2}=-f\left(r\right)dt^{2}+\frac{dr^{2}}{f(r)}+r^{2}d\phi^{2}\;,$$

where the coordinates are defined within the intervals t,r∈]−∞,+∞[ and ϕ∈[0,2π].

A particular solution to the corresponding field equations was obtained in [13], which can be represented as follows:

(4) $$f\left(r\right)=\frac{L^{2}r^{4}-MLr^{2}+2\xi }{Lr^{2}+2\xi },$$

where *M* is a real integration constant, which is interpreted as the mass of the black hole, and ξ is a real constant related to the coupling of the theory. The corresponding scalar field is given as $\varphi =\sqrt{2\sqrt{\left|\xi \right|}}\phi$. The above solution can be interpreted as a generalization of the BTZ black hole spacetime, which is contained as the limiting case ξ=0.

The outer horizon $r_{+}$ of the solution (4) is determined by the largest root of the equation f(r)=0, which is the norm of the timelike Killing associated with the time coordinate *t*. We obtain the following:

(5) $$r_{+}={\left[\frac{1}{2L}\left(M+\sqrt{M^{2}-8\xi }\right)\right]}^{1/2}.$$

The main goal of this work is to investigate the properties of the above black hole solution from the point of view of thermodynamics by using the theory of GTD. This will be done in the following sections.

## 3. Black Hole Thermodynamics

In classical thermodynamics, there are two equivalent methods to describe a given thermodynamic system. The first one consists of determining all the equations of state of the system, which essentially relates the intensive thermodynamic variables with the extensive ones. This method is especially used in applied physics and chemistry, where the equations of state are usually obtained by applying empirical procedures. The second way consists of specifying the fundamental equation that relates the extensive variables of the system and from which the equations of state can be derived by using the first law of thermodynamics [19]. Black hole thermodynamics is based on the second method.

The fundamental equation of black hole thermodynamics is the Bekenstein–Hawking formula [20,21,22,23], which relates the entropy of the black hole to the area of the corresponding horizon, i.e.,

(6) $$S=\frac{k_{B}}{4l_{P}^{2}}A_{h}\;,$$

where $k_{B}$ is the Boltzmann constant, and $l_{P}$ the Planck constant. In this work, we use geometric units, with $k_{B}=1$ and $l_{P}=1$. Furthermore, it is assumed that the first law of thermodynamics is satisfied. Consider, for instance, the Kerr black hole with mass *M* and angular momentum *J*. The computation of the entropy yields [23]

(7) $$S=2\pi \left(M^{2}+\sqrt{M^{4}-J^{2}}\right)\;,$$

which is a function of the thermodynamic variables *M* and *J*. The first law of thermodynamics in the mass representation can be expressed as follows:

(8) dM=TdS+ΩdJ,

where *T* is the Hawking temperature of the black hole, and Ω is the angular velocity at the horizon. Then, the equations of state of the black hole can be derived from the first law as follows:

(9) $$T=\frac{\partial M}{\partial S},\;\Omega =\frac{\partial M}{\partial J}\;,$$

which can be calculated explicitly by inverting Equation (7) as follows:

(10) $$M={\left(\frac{S}{4\pi }+\frac{\pi J^{2}}{S}\right)}^{1/2}\;.$$

Then, we obtain

(11) $$T=\frac{S^{2}-4\pi^{2}J^{2}}{4S\sqrt{\pi S(S^{2}+4\pi^{2}J^{2})}}\;,\;\Omega =\frac{2\pi^{3/2}J}{\sqrt{S(S^{2}+4\pi^{2}J^{2})}}.$$

An important identity that relates all the thermodynamic variables of a system is the Smarr formula [24],

(12) $$TS+\Omega J=\frac{1}{2}M,$$

which is essentially a consequence of the Euler identity of classical thermodynamics [23].

In the above description, the thermodynamic potential of the Kerr black hole is the mass *M*. It is possible to introduce alternative potentials by using Legendre transformations of *M*. In particular, we can introduce the enthalpy, Helmholtz energy, and the Gibbs free energy as follows:

(13) H(S,Ω)=M−ΩJ,F(T,J)=M−TS,G(T,Ω)=M−TS−ΩJ,

respectively. The role of these potentials in ordinary thermodynamics consists of interchanging variables for facilitating measurements. In the case of black holes, however, it is not always easy to replace the coordinates. Consider, for instance, the Gibbs free energy G(T,Ω). Using the Smarr Formula (12), we obtain

(14) $$G\left(T,\Omega \right)=\frac{1}{2}M\left(S\left(T,\Omega \right),J\left(T,\Omega \right)\right),$$

implying that the functions S=S(T,Ω) and J=J(T,Ω) should be known explicitly to be able to use G(T,Ω) as a convenient thermodynamic potential. In the case of the Kerr black hole, it is not possible to find analytic expressions for *S* and *J* in terms of *T* and Ω. For this reason, usually, the free-energy analysis is performed for the function G(S,J), which is, in fact by virtue of (14), equivalent to the analysis of M(S,J) (see, for instance, [25]). Consequently, the phase transition structure, which should be investigated by using the free energy G(T,Ω), is analyzed by using the potential M(S,J) and the corresponding heat capacities [23].

As we will see below, the theory of geometrothermodynamics is free of these difficulties because it is Legendre invariant, meaning that the analysis of any thermodynamic system can be performed independently of the thermodynamic potential and of the variables on which this potential depends.

## 4. The Theory of Black Hole Geometrothermodynamics

According to the fundamentals of GTD [26], to investigate the properties of a system from the thermodynamic and geometric points of view, it is necessary to have the explicit form of the fundamental equation. As mentioned in Section 3, in the case of black holes, the fundamental equation is determined by the Bekenstein–Hawking entropy equation that relates the entropy *S* with the surface area of the black hole horizon $A_{h}$ as $S=\frac{1}{4}A_{h}$ [20,21,22,23]. In the case of 3D gravity, the horizon area is proportional to the horizon radius so that $S=4\pi r_{+}$. Then, the entropy becomes

(15) $$S={\left[\frac{1}{L}\left(M+\sqrt{M^{2}-8\xi }\right)\right]}^{1/2},$$

where, for simplicity, we have normalized the entropy as $S\to S/(2\sqrt{2}\pi )$. We see that the entropy is a function of the cosmological constant *L* and the mass *M*.

The geometric approaches to equilibrium thermodynamics [27] consist of introducing the concept of equilibrium space E as the set of points at which the system can be in equilibrium. In the case of the fundamental Equation (15), the equilibrium states are characterized by specific values of *M* and *L* related through the fundamental Equation (15). Thus, we can use *M* and *L* as coordinates of E and, in fact, one can introduce a metric $g_{ab}^{H}$ as the Hessian of the entropy to describe the geometry of E. This approach has been applied, among others as well, in black hole thermodynamics [28]. The theory of GTD is different because it can be used with any thermodynamic potential, without changing the properties of the system. This means that we can start from the potential *S* and apply Legendre transformations to obtain new thermodynamic potentials (Massieu potentials). Classical thermodynamics is invariant with respect to Legendre transformations. This fact is used in GTD as an important ingredient, which is why GTD is called a Legendre invariant theory.

To formulate GTD for any system and any thermodynamic potential, we introduce the notation Φ for the potential and $E^{a}$ for the coordinates of E so that $\Phi =\Phi (E^{a})$ is an expression that represents the fundamental equation, in general. With this notation, the first law of thermodynamics reads $d\Phi =\sum_{a}I_{a}dE^{a}$, where $I_{a}=\frac{\partial \Phi }{\partial E^{a}}$ and a=1,…,n, with *n* being the number of thermodynamic degrees of freedom of the system. To represent Legendre transformations in such a way that they can be applied to geometric quantities, i.e., as coordinate transformations, GTD introduces the concept of the phase space T with coordinates $Z^{A}=\left\{\Phi ,E^{a},I_{a}\right\}$. Legendre invariance is then obtained by considering only objects that do not change under the coordinate transformations, which represent the Legendre transformations. The phase space is then defined as a Riemannian manifold T with a Legendre invariant metric $G_{AB}$. It has been demonstrated that, in GTD, there exist three different classes of Legendre invariant metrics whose line elements can be expressed as follows [29,30,31,32,33,34]:

(16) $$G^{I}={\left(d\Phi -I_{a}dE^{a}\right)}^{2}+\left(\beta_{ab}E^{a}I^{b}\right)\left(\delta_{cd}dE^{c}dI^{d}\right)\;,$$

(17) $$G^{II}={\left(d\Phi -I_{a}E^{a}\right)}^{2}+\left(\beta_{ab}E^{a}I^{b}\right)\left(\eta_{cd}dE^{c}dI^{d}\right)\;,$$

(18) $$G^{III}={\left(d\Phi -I_{a}dE^{a}\right)}^{2}+\sum_{a=1}^{n}\beta_{a}E^{a}I^{a}dE^{a}dI^{a}\;,$$

where $\delta_{ab}=\mathrm{diag}\left(1,1,\cdots ,1\right)$, $\eta_{ab}=\mathrm{diag}\left(-1,1,\cdots ,1\right)$, $\beta_{ab}=\mathrm{diag}\left(\beta_{1},\cdots ,\beta_{n}\right)$, and $\beta_{a}$ are the quasi-homogeneity coefficients of the fundamental equation, i.e., the constants that satisfy the condition

(19) $$\Phi \left(\lambda^{\beta_{a}}E^{a}\right)=\lambda^{\beta_{\Phi }}\Phi \left(E^{a}\right)$$

for real values of λ and $\beta_{a}$. For the particular case $\beta_{a}=1$ and $\beta_{\Phi }=1$, the above condition defines homogeneous functions of degree one, which are commonly used in ordinary thermodynamics. The coefficients $\beta_{a}$ are important because they determine the Euler identity in the following form [30]:

(20) $$\sum_{a=1}^{n}\beta_{a}I^{a}E^{a}=\beta_{\Phi }\Phi \;,$$

which is used in GTD to explore the the phase transition structure of thermodynamic systems.

Note that the only difference between the line elements $G^{I}$ and $G^{II}$ is the signature in the second term. In fact, the corresponding metrics are invariant with respect to total Legendre transformations. On the other hand, the metric components $G_{AB}^{III}$ are invariant with respect to partial and total Legendre transformations.

In the case of black holes with the fundamental equation given in Equation (15), the above Legendre invariant line elements reduce to

(21) $$G^{I/II}=G^{\pm }={\left(dS-I_{M}dM-I_{L}dL\right)}^{2}+\left(\beta_{M}I_{M}M+\beta_{L}I_{L}L\right)\left(\pm dI_{M}dM+dI_{L}dL\right)\;,$$

(22) $$G^{III}={\left(dS-I_{M}dM-I_{L}dL\right)}^{2}+\beta_{M}I_{M}MdI_{M}dM+\beta_{L}I_{L}LdI_{L}dL\;,$$

where $I_{M}$ and $I_{L}$ are the variables dual to *M* and *L*, respectively. It is easy to see that these metrics are non-degenerate, det(G)≠0, and are invariant with respect to Legendre transformations, when written in terms of the coordinates $S,M,L,I_{M},I_{L}$ as described in [26]. The phase space T is five-dimensional for the black hole under consideration, and the corresponding curvature tensors are, in general, different from zero, implying that, in all the cases, the phase spaces are curved. The construction of the phase space represents the main step in the GTD theory because it is used to explicitly impose the condition of Legendre invariance at the level at the GTD metrics.

Another important constituent of the GTD theory is the equilibrium space E. As mentioned before, the points of E can be interpreted as states of equilibrium of the corresponding thermodynamic system. This is guaranteed by demanding that, on E, the fundamental equation $\Phi =\Phi (E^{a})$ is fulfilled, which, in turn, should satisfy the laws of thermodynamics. In particular, the first law in the form $d\Phi =\sum_{a}I_{a}dE^{a}$ should be satisfied. In other words, one can say that, in GTD, a thermodynamic system is represented geometrically by the equilibrium space. An advantage of the GTD theory is that the properties of the equilibrium space E can be completely derived from the properties of the phase space T.

Indeed, in GTD, E is defined as a subspace of T determined by a smooth mapping φ:E→T, such that

(23) $$dS=I_{M}dM+I_{L}dL\;,$$

which means that the first law of thermodynamics is satisfied on E and that

(24) $$I_{M}=\frac{\partial S}{\partial M},\;I_{L}=\frac{\partial S}{\partial L}.$$

The last identities imply that $I_{M}=I_{M}\left(M,L\right)$ and $I_{L}=I_{L}\left(M,L\right)$ so that the metrics of the phase space can be reduced to two-dimensional metrics for the equilibrium space by using the relationship

(25) $$dI_{M}=\frac{\partial^{2}S}{\partial M^{2}}dM+\frac{\partial^{2}S}{\partial M\partial L}dL$$

and

(26) $$dI_{L}=\frac{\partial^{2}S}{\partial L^{2}}dL+\frac{\partial^{2}S}{\partial M\partial L}dM.$$

Following this procedure, we obtain the following line elements for the metrics of the equilibrium space

(27) $$g^{I}=F\left(\frac{\partial^{2}S}{\partial M^{2}}dM^{2}+2\frac{\partial^{2}S}{\partial M\partial L}dMdL+\frac{\partial^{2}S}{\partial L^{2}}dL^{2}\right),$$

(28) $$g^{II}=F\left(-\frac{\partial^{2}S}{\partial M^{2}}dM^{2}+\frac{\partial^{2}S}{\partial L^{2}}dL^{2}\right),$$

(29) $$g^{III}=\beta_{M}M\frac{\partial S}{\partial M}\frac{\partial^{2}S}{\partial M^{2}}dM^{2}+F\frac{\partial^{2}S}{\partial M\partial L}dMdL+\beta_{L}L\frac{\partial S}{\partial L}\frac{\partial^{2}S}{\partial L^{2}}dL^{2},$$

where

(30) $$F=\beta_{M}M\frac{\partial S}{\partial M}+\beta_{L}L\frac{\partial S}{\partial L}\;.$$

We see that the geometric properties of the equilibrium space are described by the above metrics. It is expected that each of them represents particular thermodynamic properties of the system described by the fundamental equation S=S(M,L). In general, the above expressions can be used to compute the metrics of the GTD for any system with two thermodynamic degrees of freedom. It is only necessary to identify the corresponding thermodynamic potential *S* and the independent variables *M* and *L*.

## 5. The Equilibrium Space of the 3D Regular Black Hole

As explained in the previous section, the metrics of the equilibrium space depend on the quasi-homogeneity coefficients of the thermodynamic system. Therefore, we now analyze the fundamental Equation (15) as a quasi-homogeneous function. It is easy to see that the Euler identity is not valid unless the constant ξ in (15) is considered a thermodynamic variable. Then, the condition (19) reads

(31) $$S\left(\lambda^{\beta_{M}}M,\lambda^{\beta_{L}}L,\lambda^{\lambda_{\xi }}\xi \right)=\lambda^{\beta_{S}}S\left(M,L,\xi \right)\;,$$

which is satisfied if

(32) $$\beta_{\xi }=2\beta_{M}\;,\;\beta_{S}=\frac{1}{2}\left(\beta_{M}-\beta_{L}\right)\;.$$

Taking into account these conditions, it can be verified that the Euler identity (20) in the form

(33) $$\beta_{M}M\frac{\partial S}{\partial M}+\beta_{L}L\frac{\partial S}{\partial L}+\beta_{\xi }\xi \frac{\partial S}{\partial \xi }=\beta_{S}S$$

is satisfied. The conditions (32) should be taken into account when computing the metrics of the GTD theory for the equilibrium space E. Note that this identity can be used to correctly handle the factor *F* that enters the GTD metric. Indeed, since in this particular case, we are not considering in the fundamental equation the dependence on the constant ξ, we can set $\beta_{x}i=0$ in the Euler identity only if it agrees with the conditions of quasi-homogeneity (33). In fact, we see that this choice is only possible if we also set $\beta_{M}=0$. Then, the factor *F* becomes

(34) $$F=\beta_{L}L\frac{\partial S}{\partial L}=-\frac{\beta_{L}}{2}S\;.$$

This relationship is very useful for performing explicit calculations.

**Figure 1:**
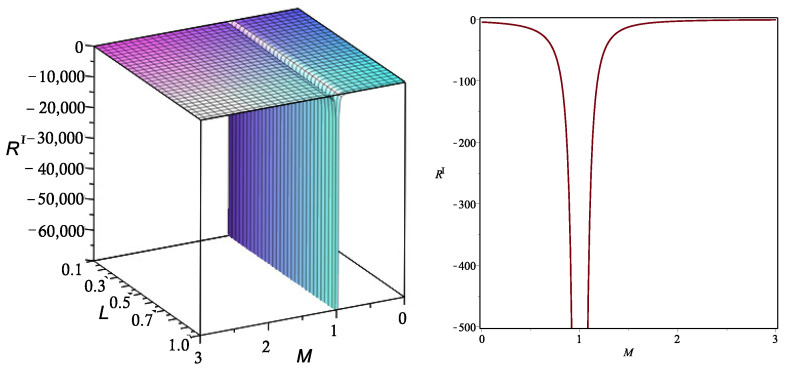
Behavior of the Ricci curvature scalar $R^{I}$ of the metric $g^{I}$ as a function of the parameters *M* and *L* (**left panel**). Illustration of the curvature singularity for the case L=1 (**right panel**). Here, for concreteness, we have chosen the particular values ξ=−1 and $\beta_{L}=1$.

We now consider the metrics for the equilibrium space. According to Equation (27), the line element $g^{I}$ can be written as

(35) $$g^{I}=\frac{\beta_{L}S^{2}}{2L^{2}}\left[\frac{M^{3}+\left(M^{2}+4\xi \right){\left(M^{2}-8\xi \right)}^{1/2}}{S^{4}{\left(M^{2}-8\xi \right)}^{3/2}}dM^{2}+\frac{LdMdL}{{\left(M^{2}-8\xi \right)}^{1/2}}-\frac{3}{2}dL^{2}\right]\;,$$

where we have used Euler’s identity in the form of (34). From here, we can calculate the explicit form of the scalar curvature, which is plotted in Figure 1 for a particular choice of parameters.

We can see that, in this case, there is a curvature singularity for a particular value of *M*, which is not affected by the value of *L*. This result indicates that, at the singular point, the black hole undergoes a phase transition. This interpretation follows from the fact that the geometric description is not valid at the points where the curvature becomes singular. From the point of view of classical thermodynamics, this corresponds to a state at which the system cannot be at equilibrium, so the approach of equilibrium thermodynamics is no more valid. This situation is usually associated with a phase transition. Then, close to the singularity, the GTD approach and equilibrium thermodynamics cannot be applied anymore.

Furthermore, the line element $g^{II}$ according to Equation (28) can be expressed as follows:

(36) $$g^{II}=\frac{-\beta_{L}S^{2}}{2L^{2}}\left[\frac{M^{3}+\left(M^{2}+4\xi \right){\left(M^{2}-8\xi \right)}^{1/2}}{S^{4}{\left(M^{2}-8\xi \right)}^{3/2}}dM^{2}+\frac{3}{2}dL^{2}\right].$$

**Figure 2:**
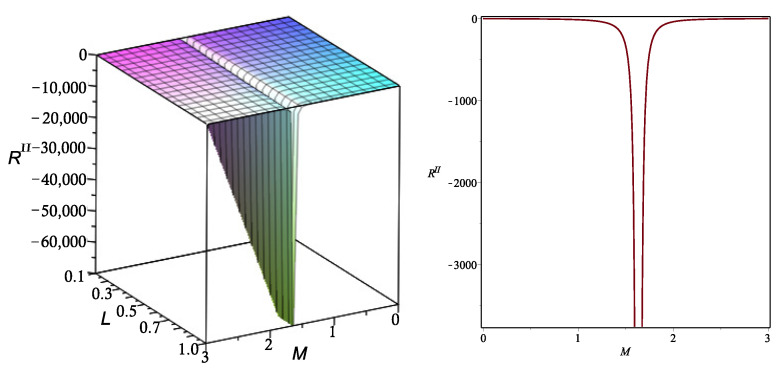
The Ricci curvature scalar $R^{II}$ of the metric $g^{II}$ as a function of the variables *M* and *L* (**left panel**). Illustration of the curvature singularity for the particular case L=1 (**right panel**). Here, ξ=−1 and $\beta_{L}=1$.

In this case, the computation of the Ricci curvature scalar is also straightforward. We show in Figure 2 the behavior of this scalar for the same choice of parameters as in the previous case.

We note again the presence of a curvature singularity for a particular value of *M* and any value of *L* in the chosen interval of parameters. This singularity indicates that a phase transition occurs as the system approaches that particular value of *M*.

Finally, from Equation (29), we obtain the following line element:

(37) $$g^{III}=\frac{\beta_{L}S^{2}}{8L^{2}}\left[\frac{L}{{\left(M^{2}-8\xi \right)}^{1/2}}dM^{2}+3dL^{2}\right]\;,$$

where we have used the Euler identity with the particular choice of quasi-homogeneous coefficients that lead to Equation (34). In this case, this choice reduces the complexity of the resulting line element in such a way that the corresponding Ricci curvature scalar vanishes, as can be shown by a direct calculation. It follows that, from the point of view of the metric $g^{III}$, no curvature singularities appear and, therefore, no phase transitions can take place.

## 6. Thermodynamic Interpretation of the Curvature Singularities

In the previous section, we found that two of the metrics of the equilibrium space lead to the appearance of curvature singularities for certain values of the mass parameter *M*. In differential geometry, singularities are interpreted as locations where geometric formalism is no longer valid, and those locations are usually eliminated from consideration due to the fact that geometric quantities such as distance cannot be defined properly. This implies that in the theory of GTD is not able to properly treat singularities, which are considered critical points where the geometric and the thermodynamic approach should break down. We will now investigate the correspondence of this breakdown on the side of thermodynamics.

In the case of the curvature scalar $R^{I}$ for the line element $g^{I}$ given in Equation (35), it turns out that it can be represented as follows:

(38) $$R^{I}=\frac{N^{I}}{{\left(D^{I}\right)}^{2}F^{3}}\;,\;D^{I}=\frac{\partial^{2}S}{\partial M^{2}}\frac{\partial^{2}S}{\partial L^{2}}-{\left(\frac{\partial^{2}S}{\partial M\partial L}\right)}^{2}\;,$$

where $N^{I}$ is a smooth function of the coordinates *M* and *L*. Then, the singularities of $R^{I}$ are determined by the conditions F=0 and/or $D^{I}=0$. The first condition cannot be satisfied by virtue of Equation (34). The second condition,

(39) $$\frac{\partial^{2}S}{\partial M^{2}}\frac{\partial^{2}S}{\partial L^{2}}-{\left(\frac{\partial^{2}S}{\partial M\partial L}\right)}^{2}=-\frac{\left(M^{2}+2\xi \right){\left(M^{2}-8\xi \right)}^{1/2}+M\left(M^{2}-2\xi \right)}{2L^{4}S^{2}{\left(M^{2}-8\xi \right)}^{3/2}}=0,$$

is satisfied for $M=\pm \sqrt{-\xi }$. We choose as singular point the positive sign in front of the squared root to be in agreement with the interpretation of *M* as the mass parameter. Then, we see that the critical value $M_{cI}=\sqrt{-\xi }$ corresponds to a curvature singularity at which the condition $D^{I}=0$ is satisfied. Interestingly, the expression $D^{I}\geq 0$ corresponds exactly to the equilibrium condition for a system with two thermodynamic degrees of freedom [19,35]. The violation of this condition $D^{I}<0$ implies that the system must pass into a state of non-equilibrium, which is usually linked with a phase transition. We conclude that the curvature singularity of the Ricci scalar $R^{I}$, which occurs at the critical value $M_{cI}=\sqrt{-\xi }$, determines the value of the mass at which the regular black hole undergoes a phase transition.

Consider now the Ricci scalar of the metric $g^{II}$ expressed in Equation (36). The calculation of the general form of $R^{II}$ shows that it can be written as

(40) $$R^{II}=\frac{N^{II}}{D^{II}}\;,\;\;D^{II}=2F^{3}{\left(\frac{\partial^{2}S}{\partial M^{2}}\frac{\partial^{2}S}{\partial L^{2}}\right)}^{2}\;,$$

where $N^{II}$ is a well-behaved function of *M* and *L*. We see that the singularities of $R^{II}$ are determined by the condition $D^{II}=0$. As mentioned before, by virtue of Equation (34), the condition F=0 is not satisfied. Then, the further condition

(41) $$\frac{\partial^{2}S}{\partial L^{2}}=\frac{3}{4L^{3}}\left(M+\sqrt{M^{2}-8\xi }\right)=0\;,$$

according to the expression of the fundamental Equation (15), cannot be satisfied because it would imply that S=0, which is not allowed from the viewpoint of thermodynamics. Finally, the condition

(42) $$\frac{\partial^{2}S}{\partial M^{2}}=-\frac{M^{3}+\left(M^{2}+4\xi \right){\left(M^{2}-8\xi \right)}^{1/2}}{2L^{2}S^{3}{\left(M^{2}-8\xi \right)}^{3/2}}=0$$

is satisfied for $M=M_{cII}=\pm \sqrt{-\frac{8}{3}\xi }$. We see that the singularity occurs when a second derivative of the fundamental equation vanishes. From the point of view of thermodynamics, these kinds of critical points are usually related to divergences at the level of the response functions [19], a result that has been also used in GTD [33]. To see this, let us consider the first law of thermodynamics in the entropy representation for the fundamental equation

(43) $$dS=I_{M}dM+I_{L}dL\;,\;\;I_{M}=\frac{S}{2L{\left(M^{2}-8\xi \right)}^{1/2}}\;,\;\;I_{L}=-\frac{S}{2L^{2}}\;.$$

The response functions are essentially represented by the change in the independent variables *M* and *L* in terms of their duals, i.e.,

(44) $$C_{L}={\left(\frac{\partial M}{\partial I_{M}}\right)}_{L}\;,\;\;C_{M}={\left(\frac{\partial L}{\partial I_{L}}\right)}_{M}\;,$$

which, by using the definition of $I_{M}$ and $I_{L}$ in terms of the derivatives of *S*, can be rewritten as

(45) $$C_{L}={\left(\frac{\partial^{2}S}{\partial M^{2}}\right)}_{L}^{-1}\;,\;\;C_{M}={\left(\frac{\partial^{2}S}{\partial L^{2}}\right)}_{M}^{-1}\;.$$

It then follows that the critical point $M_{cII}$, at which the scalar $R^{II}$ becomes singular, corresponds to a divergence in the response function $C_{L}$, which is classified as a second-order phase transition. We also see that the response function $C_{M}$ has no divergences.

**Figure 3:**
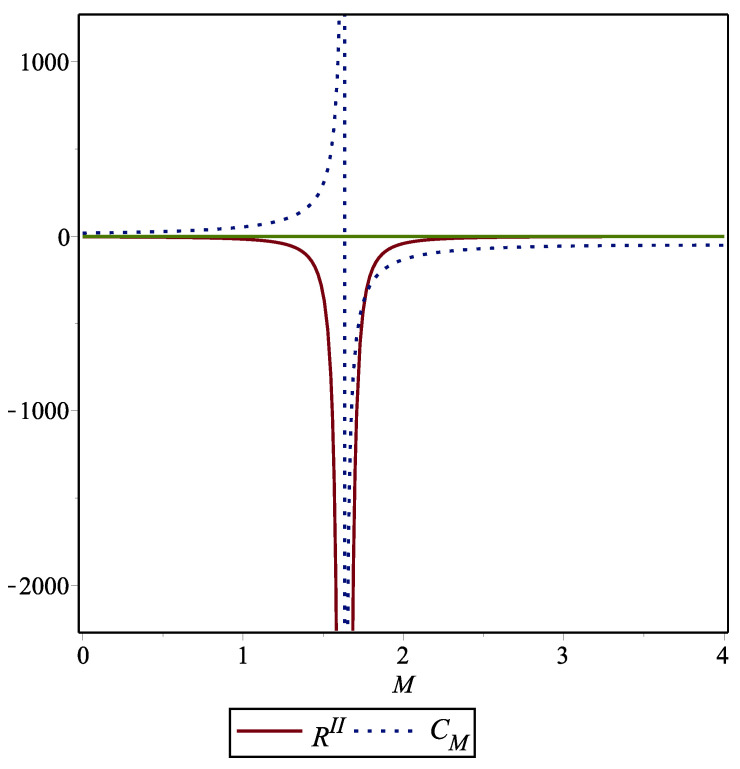
Behavior of the curvature scalar $R^{II}$ and the heat capacity $C_{M}$ as functions of the parameter *M* for the particular choices L=1, ξ=−1, and $\beta_{L}=1$.

To illustrate the fact that the curvature singularity corresponds to a phase transition, we analyze the behavior of the Ricci scalar $R^{II}$ and the heat capacity $C_{M}$ for a particular choice of parameters. The result given in Figure 3 shows clearly that the location of the curvature singularity coincides with the place where the heat capacity diverges, indicating the presence of a second-order phase transition.

## 7. Remarks and Conclusions

In this work, we have analyzed a particular solution in (2 + 1) gravity with a cosmological constant, non-minimally coupled to a real scalar field. As a result of the inclusion matter components, represented by the cosmological constant and the scalar field, the resulting theory is non-trivial as a theory of gravity, in contrast to the 3D vacuum-limiting case, which only has topological degrees of freedom.

We consider in this theory a representative spherically symmetric black hole solution that is free of curvature singularities in the entire spacetime. We calculate the value of the outer event horizon and use the Bekenstein–Hawking entropy relation to determine the entropy of the black hole, which corresponds to the fundamental equation from the point of view of thermodynamics.

We then investigate the properties of this black hole from the point of view of GTD. First, we compute the Legendre invariant metrics for the phase space, which turns out to be a 5D Riemannian manifold. To this end, we first analyze the fundamental equation as a quasi-homogeneous function and determine the relations between the coefficients of quasi-homogeneity, which explicitly enter the expressions of the Legendre invariant metrics. Then, we compute the explicit forms of the metrics of the space of the equilibrium state, which is a 2D Riemannian manifold with coordinates *M* and *L*. Furthermore, we perform a numerical analysis of the behavior of the corresponding curvature scalars, finding that there are two curvature singularities for some particular values of the mass parameter *M*.

To find out the significance of the thermodynamic singularities, we investigate curvature scalars analytically. Our results show that, in the equilibrium space of the black hole, in general, there are two curvature singularities located at $M=M_{cI}=\sqrt{-\xi }$ and $M=M_{cII}=\sqrt{-\frac{8}{3}\xi }$. The first one corresponds to a violation of the equilibrium condition, whereas the second one is a second-order phase transition. We see that singularities exist only for negative values of the coupling constant ξ, implying that regular black holes with positive values of ξ are also regular from the point of view of GTD.

We conclude that the particular 3D regular black hole analyzed in this work has a rich thermodynamic structure in which, depending on the value of the coupling constant ξ, there are two completely different cases. In the first case, with a positive coupling constant, the black hole is thermodynamically active, in the sense that the space of equilibrium states is curved, but with a completely regular behavior from the point of view of its phase transition structure. The second case, with a negative coupling constant, consists of a regular black hole with a singular space of equilibrium states, where the singularities correspond to non-equilibrium states that cannot be described either by classical thermodynamics or by GTD.

Note that the GTD analysis presented in this work can be performed in a similar way to any black hole with two thermodynamic degrees of freedom, which are the independent variables $E^{a}$ that appear in the fundamental equation $\Phi =\Phi (E^{a})$. In fact, the interpretation of the variables $E^{a}$ as thermodynamic variables is important because it allows us to pass from the thermodynamic approach to an effective statistical approach, from which information about the microscopic degrees of freedom of black holes can be obtained. This is an interesting result of using thermodynamics and geometrothermodynamics in black hole physics [36,37,38,39].

## Data Availability

No new data were created or analyzed in this study. Data sharing is not applicable to this article.
